# Editorial: Novel regulatory mechanisms behind thermogenesis of brown and beige adipocytes, volume II

**DOI:** 10.3389/fendo.2024.1510212

**Published:** 2024-10-29

**Authors:** Rini Arianti, Abhirup Shaw, Endre Kristóf, Rubén Cereijo

**Affiliations:** ^1^ Laboratory of Cell Biochemistry, Department of Biochemistry and Molecular Biology, Faculty of Medicine, University of Debrecen, Debrecen, Hungary; ^2^ Rosalind & Morris Goodman Cancer Institute, McGill University, Montreal, QC, Canada; ^3^ Departament de Bioquímica i Biomedicina Molecular, Universitat de Barcelona, Institut de Biomedicina de la Universitat de Barcelona (IBUB), and Institut de Recerca de Sant Joan de Déu, Barcelona, Spain; ^4^ Centro de Investigación Biomédica en Red (CIBER) Fisiopatología de la Obesidad y Nutrición, Madrid, Spain

**Keywords:** obesity, brown adipocytes, beige adipocytes, UCP 1, thermogenesis, beigeing, browning

## Introduction

Brown and beige adipocytes are specialized cells that play a crucial role in systemic metabolism. Unlike white adipocytes, which primarily store energy, these cells generate heat by burning calories through non-shivering thermogenesis (1). This is facilitated by uncoupling protein 1 (UCP1), which dissipates the proton gradient within their mitochondria, in addition to UCP1-independent mechanisms (1, 2). While brown adipocytes are found in dedicated depots of brown adipose tissue (BAT), beige adipocytes lie interspersed within white adipose tissue (WAT), and can be recruited in response to cold or hormonal signals in a process known as browning or beigeing (1). Both cell types can also release batokines, signaling molecules coordinating systemic metabolic responses (3). Given these unique properties, beige and brown adipocytes lie at the forefront of out-of-the-box strategies to counter obesity and associated comorbidities like diabetes. This Research Topic aimed to understand their regulatory mechanisms, crucial to eventually harness their beneficial systemic metabolic actions in human healthcare.

## Novel mechanisms regulating browning and thermogenic activation

Understanding the involvement of relevant regulators of the thermogenic transcriptional program is of utmost relevance to benefit from the metabolism-orchestrating functions of brown and beige adipocytes. In this regard, Nie et al. comprehensively reviewed this question’s state of the art from different points of view. These include the regulation of browning through norepinephrine-independent mechanisms mediated by secreted factors like novel autocrine batokine ADISSP and membrane receptors (GPR180), control by transcription factors (OVOL2) and the roles of enzymes and other cell dynamics regulators, such as mitochondrial cristae biogenesis regulator OPA1. They also highlight the activating influence of environmental factors on brown/beige adipocyte biology, notably Covid-19 and Zn.

Cold-induced thermogenesis and beigeing are primarily regulated by the activation of sympathetic nervous system which densely innervates BAT. The released norepinephrine binds to β_3_-adrenergic receptors, which induce a signaling cascade mediated via adenylyl cyclase activation by Gs proteins (1). Benzi et al. describe another critical regulator of thermogenic activation. Transient receptor potential cation channel, subfamily M, member 2 (TRPM2)-deficient mice are cold intolerant because of blunted BAT activation and WAT beiging. TRPM2 is a Ca^2+^ channel gated by adenosine diphosphate ribose (ADPR), which is produced at a greater extent in response to cold.

Beigeing relies on the activation of specific gene expression programs that are coordinately regulated by a set of unique transcriptional and epigenetic regulators which drive adipose-selective chromatin architectures (4). Mooli et al. identify GA-binding protein alpha (GABPα) as a critical transcriptional regulator essential for beige adipogenesis in inguinal WAT in the postnatal period of mice. The GABPα binding motif is enriched in epigenetically active chromatin regions, such as *Ucp1* enhancer, marked by acetylated histone 3 lysine 27 (H3K27ac) in inguinal WAT.

Accumulating recent evidence also points toward splicing regulation and alternative splicing as emerging key mechanisms in brown/beige adipogenesis and activation (5, 6). Coherently, expression of splicing factor SF3B1 has been reported as instrumental in murine brown adipocyte thermogenic activation (7). In this Research Topic, Pickering et al. explore the regulation of alternative splicing in human beige adipocytes by conducting high-throughput RNA-sequencing. The authors identify a differential alternative splicing profile between white and beige adipocytes derived from pluripotent adipose stromal cells, including beigeing markers (*CITED1*), metabolically-relevant enzymes and, notably, master adipogenic transcription factor peroxisome-activator proliferator gamma (*PPARG*). Altogether, these findings indicate that alternative splicing adds a novel layer of regulation to human beige adipogenesis.

## Innovative experimental models to study brown/beige adipocytes

*Ex vivo* differentiated primary adipocytes have been widely used to understand the adipose tissue biology and metabolism. However, current 2D models do not demonstrate the complex microenvironment and vasculature of adipose tissues. As an alternative, 3D cultures represent the cellular crosstalk and physiology in adipose tissue and provide a less-invasive method with high reproducibility (8). Davidsen et al. present a novel *in vitro* model utilizing vascularized adipose spheroids derived from mouse inguinal WAT to study thermogenic adipocyte metabolism and microenvironment in-depth. This spheroid model mimics the natural organization and vascularization of adipocytes, marked by the appearance of adipocyte capillary structures and vessel formation. Additionally, vascularized adipose spheroids showed higher expression of genes involved in metabolic pathways suggesting that 3D spheroid model may become a more detailed and accurate *in vitro* platform to study the potential of thermogenic adipocytes as a therapeutic strategy to combat obesity and related diseases.

## Thermogenic adipocyte inter-organ communication

Skeletal muscles and intestinal microorganisms play an important role in generating metabolites which can affect the metabolic activity in the body (9, 10). Tang et al. wrote a comprehensive review exploring the intricate relationship between skeletal muscles and intestinal-derived metabolites and adipocyte thermogenesis. Skeletal muscles release lactate, kynurenic acid, inosine, and β- aminoisobutyric acid, whereas the gut secretes bile acids, butyrate, succinate, cinnabarinic acid, urolithin A, and asparagine. These metabolites can mediate thermogenesis by interacting with membrane receptors or enzymes thus promoting cellular signaling. In addition, these metabolites also play role as mediators of inter-organ crosstalk and promote metabolic adaptability.

Cardiovascular diseases are leading causes of global morbidity and mortality. Perivascular adipose tissue (PVAT) is a specific type of adipose tissue that encircles blood vessels with particular secretory functions (11, 12). In their review, Tong et al. provided an interesting overview on the importance of PVAT in cardiovascular health. They summarize the origin, organization, and tissue structure of PVAT, as a combination of WAT and BAT, the proportion of which varies regarding the proximity to the organs involved. PVAT mediates vasoprotective effects by releasing adipocyte-derived relaxing factors (ADRFs), which include adiponectin, leptin, nitric oxide, hydrogen sulfide, hydrogen peroxide, and fibroblast growth factor-21. Hence, PVAT has immense potential as a therapeutic target for restoring, delaying, and counteracting vascular dysfunction.

## Closing remarks and future perspectives

This Research Topic, alongside Part I (13), has provided remarkable original research and reviews, adding novel insights to the intricate role of BAT and beiging in metabolic homeostasis. Fully understanding the regulatory mechanisms governing brown/beige adipogenesis and activation is critical to develop new therapeutic approaches against obesity and concomitant metabolic alterations and improve metabolic human health.
